# Impact of quality of response on survival outcomes among multiple myeloma patients treated with novel agents – a retrospective analysis

**DOI:** 10.1590/1516-3180.2021.0174.R2.22062021

**Published:** 2022-01-21

**Authors:** Irena Ćojbašić, Miodrag Vučić, Ivan Tijanić, Žarko Ćojbašić

**Affiliations:** I MD, PhD. Assistant Professor, Department of Internal Medicine, Faculty of Medicine, University of Niš, Niš, Serbia; and Hematologist, Clinic of Hematology and Clinical Immunology, University Clinical Centre Niš, Niš, Serbia.; II MD, PhD. Associate Professor, Department of Internal Medicine, Faculty of Medicine, University of Niš, Niš, Serbia; and Hematologist, Clinic of Hematology and Clinical Immunology, University Clinical Centre Niš, Niš, Serbia.; III MD, PhD. Assistant Professor, Department of Internal Medicine, Faculty of Medicine, University of Niš, Niš, Serbia; and Hematologist, Clinic of Hematology and Clinical Immunology, University Clinical Centre Niš, Niš, Serbia.; IV PhD. Full Professor, Department of Mechatronics and Control, Faculty of Mechanical Engineering, University of Niš, Niš, Serbia.

**Keywords:** Multiple myeloma, Therapeutics, Risk factors, Myeloma multiplex, Treatment, Outcome

## Abstract

**BACKGROUND:**

In this era of target therapies, novel data on the correlation between response endpoints and survival outcomes in multiple myeloma have arisen.

**OBJECTIVE:**

To determine the impact of quality of response on clinical outcomes, using first-line treatment, and identify risk factors influencing progression-free survival (PFS) and overall survival (OS) among myeloma patients.

**DESIGN AND SETTING:**

Retrospective analysis on myeloma patients who were treated at the Clinic of Hematology and Clinical Immunology, University Clinical Centre, Niš, Serbia, over a four-year period.

**METHODS:**

A total of 108 newly diagnosed patients who received first-line therapy consisting of conventional chemotherapy or novel agent-based regimens were included in this analysis.

**RESULTS:**

The quality of response to first-line therapy for the whole cohort was classified as follows: complete response (CR) in 19%; very good partial response (VGPR) in 23%; partial response (PR) in 38%; and less than PR for the remaining patients. After a median follow-up of 25.4 months, the three-year PFS and OS for the entire study population were 47% and 70%, respectively. Achievement of CR was the main factor associated with significantly prolonged PFS and OS, in comparison with patients who reached VGPR and PR. Likewise, addition of the new drugs bortezomib and thalidomide to standard chemotherapy led to considerably extended PFS and OS, compared with conventional therapy alone.

**CONCLUSIONS:**

This analysis demonstrated that the quality of response after application of first-line treatment using novel agent-based regimens among multiple myeloma patients was a prognostic factor for PFS and OS, which are the most clinically relevant outcomes.

## INTRODUCTION

Multiple myeloma (MM) is an incurable plasma cell neoplasm exemplified by changeable survival ranging from several months to more than 15 years. The prognosis can be impacted by disease biology, type of therapy, quality of response and patient-related factors. Several recent studies have shown improved outcomes for patients with myeloma, regarding both relapses of the disease and diagnoses, through treatments with new myeloma-directed drugs, autologous stem cell transplants and combination therapeutic approaches.^1,2^

Enhancing long-term outcomes is the primary aim of current treatment strategies, including overall survival (OS) and progression-free survival (PFS). First-line treatment response is one of the most crucial prognostic factors related to PFS and OS among patients with newly diagnosed MM.^3^ Use of a combination of thalidomide, melphalan and prednisone as first-line treatment in patients with multiple myeloma, in comparison with melphalan-prednisone, was shown to provide significant improvements, both in PFS (15.0 versus 11.0 months) and in OS (two-year OS rates were 67% versus 43%).^4^ The time to progression among patients receiving bortezomib plus melphalan-prednisone was significantly longer than the time among those receiving melphalan-prednisone alone (24.0 versus 16.6 months).^5^ Moreover, the VISTA study confirmed longer OS (three-year OS rates were 68.5% versus 54.0%) and other clinical benefits through use of bortezomib plus melphalan-prednisone versus use of melphalan-prednisone.^6^ Assessment on the influence of the degree of treatment response on PFS and OS showed that the three-year PFS and OS were significantly prolonged among patients who achieved complete response (CR), compared with those who achieved very good partial response (VGPR) or partial response (PR).^7^ Data from a meta-analysis indicated that achievement of CR subsequent to high-dose chemotherapy (HDT) and autologous stem cell transplantation (ASCT), after first-line therapy with novel versus non-novel agents, has more prognostic influence for enhanced long-term outcomes.^8^

## OBJECTIVE

The aim of this study was to assess the impact of the quality of therapeutic response with first-line treatment on progression-free survival and overall survival, among newly diagnosed myeloma patients, along with extensive analysis on prognostic factors in relation to survival outcomes.

## METHODS

Consecutive newly diagnosed MM patients who were treated at our institution over the period from January 2015 to December 2018 were retrospectively evaluated. This study included patients whose response data after first-line therapy were available. The diagnosis of MM was determined in accordance with the updated criteria of 2014 from the International Myeloma Working Group (IMWG).^3^ Staging and risk assessment were done in accordance with the international staging system for multiple myeloma.^9^

Patients were treated either with conventional chemotherapy or with novel agent-based induction comprising regimens that included either thalidomide or bortezomib. Therapeutic response assessment was carried out in accordance with the IMWG consensus response criteria.^10,11^

Briefly, CR was defined as negative serum and urine immunofixation, presence of less than 5% plasma cells in bone marrow and disappearance of any soft tissue plasmacytoma. VGPR was characterized as a reduction in serum M-protein of 90% or more; and urinary M-protein of less than 100 mg/24 hours or M-protein noticeable through immunofixation but not through electrophoresis. Partial response (PR) was defined as a reduction in serum M-protein levels from baseline of 50% or more; and a reduction in 24-hour urine M-protein excretion of 90% or more, or a level of less than 200 mg/24 hours. The disease was classified as stable if did not fulfil the criteria for PR, VGPR, CR or progressive disease. Any of the following was defined as progressive disease: an increase of 25% or more from the lowest response value in serum M-protein (absolute ≥ 0.5 g/dl) or urine M-protein (absolute ≥ 200 mg/24 hours).

OS was estimated from the time of diagnosis until death or the last follow-up. PFS was calculated from the time of diagnosis to disease progression, relapse or death from any cause or the last follow-up.

The Pearson χ^2^ test was used to compare patient characteristics regarding discrete variables while the Mann-Whitney test was applied to continuous variables. The prognostic influence of therapeutic and clinical factors on PFS and OS was assessed based on the hazard ratio (HR) with 95% confidence interval (Cl) from multivariate Cox’s proportional hazards regression. All statistical tests were two-sided, and P-values < 0.05 were considered statistically significant. Survival analysis, regarding PFS and OS, was performed by applying the Kaplan-Meier method. Survival outcomes were analyzed for patients who achieved CR, VGPR or PR after induction therapy.

This study was approved by our institution’s ethics committee (date: January 19, 2021; number: 1399/2).

## RESULTS

This retrospective analysis included 108 patients who were newly diagnosed with multiple myeloma. Their median age at diagnosis was 63.8 years (range 40-82 years), and 53% were males. At the time of diagnosis, the majority of the patients (62; 57%) were in clinical stage III of Durie & Salmon,^12^ while 52 patients (48%) had high-risk disease.

The number of patients who initially had substantial renal impairment with creatinine clearance (CrCl) < 40 ml/minute was 26 (24%), while 12 (11%) patients were undergoing hemodialysis. More than half of the patients had Charlson’s comorbidity index (CCI) ≥ 2, and a smaller percentage of these patients achieved CR and VGPR, compared with patients who had CCI < 2. Equal distribution between international staging system (ISS) stages I, II and III was recorded among patients in the CR, VGPR and PR groups; this equal distribution was also seen in relation to clinical stages. The types of therapy administered and baseline characteristics of the patients who achieved CR, VGPR and PR are shown in Table 1.

**Table 1.** Baseline characteristics of patients with multiple myeloma

Variable	All patients n = 108	CR n = 20	VGPR n = 25	PR n = 41
**Sex**				
Male, n (%)	57 (53)	11 (55)	13 (52)	21 (51)
**Age, years**				
**Median**	63.8	42-72	62.7	63.3
Range	40-82	64.5	49-79	41-85
≥ 70 years, n (%)	31 (29)	4 (20)	4 (16)	9 (22)
**Charlson’s comorbidity index, n (%)**				
CCI < 2	47 (43.5)	11 (55)	15 (60)	18 (44)
CCI ≥ 2	61 (56.5)	9 (45)	10 (40)	23 (56)
**Durie & Salmon stage, n (%)**				
I	14 (13)	2 (10)	2 (8)	7 (17)
II	32 (30)	6 (30)	7 (28)	13 (32)
III	62 (57)	12 (60)	16 (64)	21 (51)
**Creatinine clearance, n (%)**				
CrCl < 40 ml/min	26 (24)	4 (20)	7 (28)	13 (32)
CrCl ≥ 40 ml/min	82 (76)	16 (80)	18 (72)	28 (68)
**ISS stage, n (%)**				
I	18 (17)	4 (20)	4 (16)	7 (17)
II	38 (35)	7 (35)	9 (36)	14 (34)
III	52 (48)	9 (45)	12 (48)	20 (49)
**Type of therapy, n (%)**				
Conventional	11 (10)	1 (5)	2 (8)	4 (10)
Thalidomide	74 (69)	11 (55)	16 (64)	32 (78)
Bortezomib	23 (21)	8 (40)	7 (28)	5 (12)

CR = complete response; VGPR = very good partial response; PR = partial response; ISS = international staging system.

Novel agent-based induction therapy was applied to 97 patients, among whom 74 received a drug combination with thalidomide and 23 received a drug combination with bortezomib. The other 11 patients underwent conventional chemotherapy. The quality of response to first-line therapy was assessed as follows: CR was reported in 20 cases (19%), VGPR in 25 (23%) and PR in 41 (38%); the remaining patients achieved less than PR. Patients who achieved CR were generally treated with novel agent-based regimens: 55% of these patients received combination therapy with thalidomide, 40% combination therapy with bortezomib and only 5% conventional chemotherapy. In contrast, in the PR group, the highest number of patients (78%) received combined therapy with thalidomide, 12% combination therapy with bortezomib and 10% standard chemotherapy.

The influence of new therapeutic modalities on the recovery of renal function and whether its recovery affected the OS was analyzed. The rate of achieving complete renal response in accordance with the IMWG criteria among patients with CrCl < 40 ml/minute at diagnosis was higher in the group of patients initially treated with bortezomib-based protocols than in the group that received thalidomide protocols: 40% versus 22% (P = 0.061). There was a significant difference in median OS in the group of patients who had CrCl < 40 ml/minute at diagnosis and corrected this to CrCl ≥ 40 ml/minute, compared with the group that continued to present CrCl < 40 ml/minute after therapy: 32.5 months versus 18.6 months (P = 0.019).

**Figure 1.**
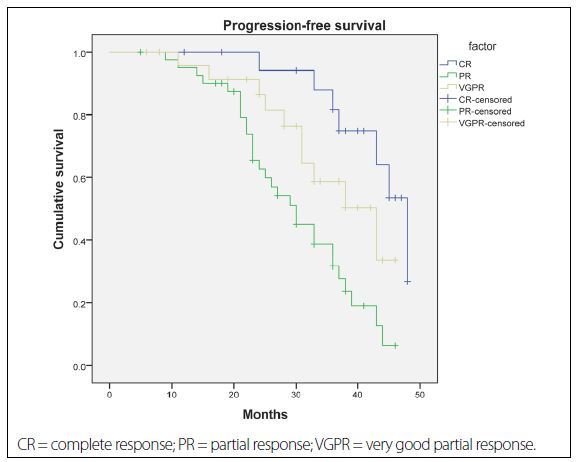
Kaplan-Meier estimate of progression-free survival according to quality of response. Estimated progression-free survival among patients receiving first-line therapy, according to quality-of-response group.

**Figure 2.**
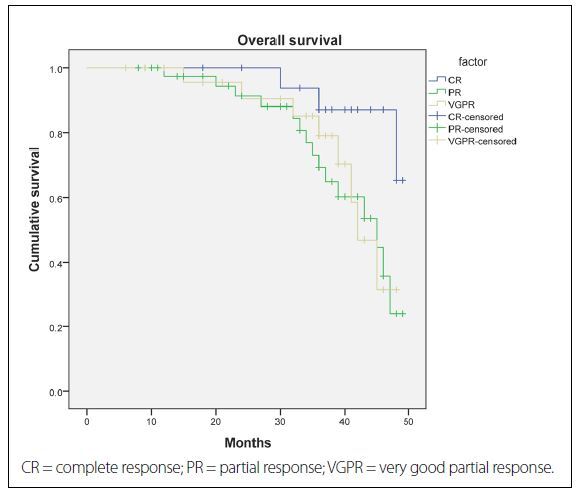
Kaplan-Meier estimate of overall survival according to quality of response. Estimated overall survival among patients receiving first-line therapy, according to quality-of-response group.

After a median follow-up of 25.4 months (range 6-48 months), the three-year PFS and OS for all the patients analyzed were 47% and 70%, respectively. The outcomes according to quality of treatment response to first-line therapy among multiple myeloma patients are shown in Figure 1 and Figure 2. The three-year PFS was 75% among patients who achieved CR after first-line treatment, 49% among patients who achieved VGPR (HR = 0.19; 95% Cl, 0.12-0.23; P < 0.001) and 32% among those who only reached PR (HR = 0.11; 95% Cl, 0.06-0.17; P < 0.001) (Figure 1). Likewise, the three-year OS was 87% among patients who achieved CR after first-line treatment, 72% among those who reached VGPR (HR = 0.17; 95% Cl, 0.10-0.27; P < 0.001) and 66% among those who only achieved PR (HR = 0.09; 95% Cl, 0.05-0.18; P < 0.001) (Figure 2).

CR = complete response; PR = partial response; VGPR = very good partial response.

CR = complete response; PR = partial response; VGPR = very good partial response.

The effects of comorbidities on OS and PFS were assessed using Charlson’s comorbidity index, in which patients with CCI < 2 were considered to be fit and patients with CCI ≥ 2, frail. In this cohort, according to CCI, fit patients accounted for 43.5% and frail for 56.5%. Fit patients were found to have higher rates for three-year OS than frail patients (80% versus 57.6%; HR = 0.13; 95% Cl, 0.05-0.37; P = 0.011). Also, the rates for three-year PFS were higher in the group of fit patients than in the group of frail patients (67% versus 24.6%; HR = 0.28; 95% Cl, 0.14-0.48; P = 0.005).

The findings from the assessment of prognostic factors that influenced survival are presented in Table 2. Multivariate analysis showed that novel agent-based induction, namely the addition of bortezomib or thalidomide to conventional chemotherapy, was linked to substantial enhancement of PFS: (HR = 0.55; 95% Cl, 0.23-0.87; P < 0.001) and (HR = 0.79, 95% Cl, 0.38-1.13, P < 0.001), respectively. Achievement of CR after first-line treatment, compared with VGPR (HR = 0.31; 95% Cl, 0.13-0.54; P < 0.001) and PR (HR = 0.22; 95% Cl, 0.10-0.46; P < 0.001), was significantly associated with prolonged PFS. Shorter PFS was related to age of more than 65 years, existence of comorbidities (CCI ≥ 2) and renal impairment that continued even after therapy (CrCl < 40 ml/minute). On the other hand, this association was not established for clinical stages II and III, or for the existence of intermediate and high-risk diseases.

**Table 2.** Multivariate analysis on factors possibly influencing progression-free survival and overall survival

Factor	PFS HR (95% Cl)	P-value	OS HR (95% Cl)	P-value
**Age, years**				
> 65 versus ≤ 65	1.18 (1.09-1.23)	0.02	1.36 (1.18-1.52)	< 0.001
**Charlson’s comorbidity index**				
CCI ≥ 2 versus CCI < 2	0.59 (0.41-0.78)	0.04	0.91 (0.64-1.21)	0.003
**ISS stage**				
2 versus 1	1.10 (0.67-1.45)	0.39	1.25 (0.89-1.65)	0.57
3 versus 1	1.32 (0.83-1.75)	0.19	1.45 (0.91-1.83)	0.10
**Durie & Salmon stage**				
II versus I	1.48 (1.02-1.78)	0.17	1.67 (1.23-1.97)	0.27
III versus I	1.40 (1.14-1.81)	0.12	1.59 (1.19-1.92)	0.09
**Creatinine clearance after therapy**				
Retained CrCl < 40 ml/min versus reversed CrCl ≥ 40 ml/min	1.29 (1.17-1.49)	0.01	1.12 (0.84-1.37)	< 0.001
**Therapy**				
Bortezomib combination versus conventional	0.55 (0.23-0.87)	< 0.001	0.42 (0.19-0.88)	< 0.001
Thalidomide combination versus conventional	0.79 (0.38-1.13)	< 0.001	0.68 (0.44-1.09)	< 0.001
**Response**				
CR versus VGPR	0.31 (0.13-0.54)	< 0.001	0.39 (0.16-0.63)	< 0.001
CR versus PR	0.22 (0.10-0.46)	< 0.001	0.27 (0.11-0.50)	< 0.001

PFS = progression-free survival; OS = overall survival; HR = hazard ratio; ISS = international staging system; CR = complete response; VGPR = very good partial response; PR = partial response.

Regarding OS, patients who received combined therapy with bortezomib (HR = 0.42; 95% Cl, 0.19-0.88; P < 0.001) or a therapeutic combination with thalidomide (HR = 0.68; 95% Cl, 0.44-1.09; P < 0.001) showed a significant association with superior survival, compared with patients treated with conventional chemotherapy. Achievement of CR was the factor most strongly correlated with significantly prolonged OS, in comparison with VGPR (HR = 0.39; 95% Cl, 0.16-0.63; P < 0.001) and PR (HR = 0.27; 95% Cl, 0.11-0.50; P < 0.001). Age greater than 65 years, presence of comorbidities (CCI ≥ 2) and absence of recovery of renal function after therapy (CrCl < 40 ml/minute) were the factors that were found to significantly reduce OS, while higher clinical stage and high-risk disease did not have any impact.

## DISCUSSION

The prognostic influence of the quality of response has been proven mainly among patients newly diagnosed with MM who were treated with HDT/ASCT. Patients who achieved a maximal response were more likely to have better long-term survival than those reaching lesser responses.

Lahuerta et al.^13^ assessed therapeutic responses among patients with newly diagnosed MM that was treated through chemotherapy induction regimens tracked by means of HDT/ASCT. There was a considerable correlation between depth of response and outcomes, such that there was a five-year OS rate of 74% among patients who achieved CR, compared with 50% for patients who achieved PR (P = 0.01). In a study by Moreau et al.,^14^ PFS was significantly longer for patients who achieved VGPR later in induction therapy, compared with patients who achieved VGPR only after HDT/ASCT (median of 41.2 versus 31.1 months; P = 0.01). This evidently suggests that achievement of VGPR or better after induction has prognostic significance for longer PFS.

A recently published meta-analysis on 24 studies among newly diagnosed myeloma patients undergoing ASCT that examined the connectivity between responses and long-term outcomes showed that the association between achieving CR and outcomes seemed to be better for patients achieving CR through use of novel rather than non-novel agents.^8^

After the introduction into clinical practice of target therapy using the proteasome inhibitor bortezomib and the immunomodulatory drugs thalidomide and lenalidomide, as part of combination treatments, these therapies have enabled greater emphasis on the depth and duration of responses and their influence on improvements in disease control and survival.^1,15^ In a study by Offidani et al.,^16^ the efficiency of thalidomide-based regimens among untreated patients with MM was assessed and was shown to provide a higher response rate, while the response to treatment was significantly predictive of survival. The estimated three-year time to progression was 60%, event-free survival was 57% and OS was 84%, and these parameters were significantly higher among patients who achieved a response level of CR/VGPR than among those who did not.

The results from the VISTA study^17^ showed that the quality of response was correlated with improved long-term outcomes among patients treated with bortezomib-based regimens. These analyses indicated that achievement of CR was correlated with substantially longer periods to progression, length of time to next therapy and greater treatment-free interval, but there was no significant difference in OS. Falcon et al.^18^ reported that patients treated with lenalidomide-based regimens had significantly longer OS than patients treated with conventional chemotherapy (59.1 versus 49.1 months; P = 0.0144). In addition, the four-year PFS rate was more than doubled in the lenalidomide group, to 32.6%, compared with 14.6% in the group treated with standard therapy (P < 0.0001), thus proving that lenalidomide-based regimens significantly improved PFS.

Gay et al.^7^ performed a pooled analysis on 1,175 newly diagnosed myeloma patients who were treated with melphalan-prednisone with or without thalidomide and/or bortezomib. The highest CR rate was detected among patients treated with bortezomib and thalidomide plus melphalan-prednisone (49%), while the lowest rate was among patients treated with melphalan-prednisone (5%), with a strong correlation between depth of response and outcomes. After a median follow-up of 29 months, the three-year PFS and OS were 67% and 27% (P < 0.001), and 91% and 70% (P < 0.001), among patients who achieved CR and those who achieved VGPR, respectively.

The present analysis demonstrated that the CR rates were similar in the groups of patients treated with thalidomide-based regimens (55%) and bortezomib-based regimens (40%), and were lowest in the group treated with conventional therapy (5%). This study also showed that after a median follow-up of 25.4 months, the three-year PFS and OS were 75% and 49% (P < 0.001), and 87% and 72% (P < 0.001), among patients who achieved CR and those who achieved VGPR, respectively. Moreover, multivariate analysis indicated that novel agent-based induction, achievement of CR after first-line treatment, recovery of renal function, absence of significant comorbidities and age less than 65 years were the factors that were clearly linked with considerably prolonged PFS and OS.

Multivariate risk factor analysis by other researchers has shown that novel agent-based induction, administration of maintenance therapy and achievement of CR were significantly linked with prolonged PFS. Regarding OS, novel agent-based induction and maintenance therapy were significantly associated with superior survival.^19^ Multidrug regimens combining proteasome inhibitors with immunomodulatory drugs have enhanced the depth of response, have shown satisfactory tolerability and are being recommended as a standard treatment approach.^20^ The rapidity of achievement of a deep response after up-front therapy, applied either early or late during the treatment, does not influence survival.^21^ Treatment should be personalized to the disease characteristics, for individual patients, with the goal of achieving better disease control and longer survival.^20^

## CONCLUSION

The findings from the present analysis indicate that the quality of response to first-line treatment has a positive prognostic impact. Patients who achieved a maximal response had significantly longer progression-free survival and overall survival, compared with patients who reached lesser responses. In our study, the patients who were treated with novel agent-based induction regimens had significantly prolonged progression-free survival and superior overall survival, in comparison with the patients who were treated with conventional chemotherapeutic agents. The data from this study support the conclusion that achievement of a deeper response after first-line treatment with novel targeted therapies, among multiple myeloma patients, is a prognostic factor for improved survival outcomes.
